# Software Authority Transition through Multiple Distributors

**DOI:** 10.1155/2014/295789

**Published:** 2014-07-20

**Authors:** Kyusunk Han, Taeshik Shon

**Affiliations:** ^1^University of Michigan, 500 S. State Street, Ann Arbor, MI 48109, USA; ^2^Department of Information Computer Engineering, Ajou University, San 5, Woncheon-dong, Yeongtong-gu, Suwon 443-749, Republic of Korea

## Abstract

The rapid growth in the use of smartphones and tablets has changed the software distribution ecosystem. The trend today is to purchase software through application stores rather than from traditional offline markets. Smartphone and tablet users can install applications easily by purchasing from the online store deployed in their device. Several systems, such as Android or PC-based OS units, allow users to install software from multiple sources. Such openness, however, can promote serious threats, including malware and illegal usage. In order to prevent such threats, several stores use online authentication techniques. These methods can, however, also present a problem whereby even licensed users cannot use their purchased application. In this paper, we discuss these issues and provide an authentication method that will make purchased applications available to the registered user at all times.

## 1. Introduction

In recent years, software distribution models have changed rapidly. Apple's* iOS Appstore* and* iTunes* made a significant change to the ecosystem of software and content distribution.

The convenience of these systems inspired other competitors and solutions. For mobile devices, for example, Google launched* Google Play* for their Android OS, and Amazon have developed their own* Amazon Appstore*. The success of these endeavors has influenced the PC-based OS software ecosystem. Microsoft has recently released* Windows Store* for Windows 8, and Apple has released* Mac Appstore*.

Whereas Apple's iOS only allows access to their built-in store, most distributors allow users other options. For example, the Android system allows access to Google's built-in store service as well as other mobile carriers' store services, even including those manually installed by the user. While users can purchase apps from the Windows and Mac appstores, they can also purchase them from other distributors or developers as well.

Although such market services provide significant convenience to users, they have introduced several issues. With the traditional software purchase environment, users could obtain product support regardless of where they purchased their applications. Users who purchase an application from a specific online appstore, however, cannot get support if they cancel or lose their connection to the store. Moreover, if an app requires an online authentication process to verify a valid license, the user will not even be able to launch the app.

In this paper, we discuss the software authorization issue and propose an extended “purchase authentication service” (PAS) model [1] that ensures users are authorized to access applications even if they change their status. Our extended PAS avoids the use of an independent system, which can cause overheads. We demonstrate two scenarios: (1) users are using a roaming service and (2) users permanently change their contact details.

We present an overview of the online application store model in Section 2. In Section 3, we discuss problems with application authorization. We then propose, in Section 4, an authentication protocol that allows a user to obtain authorization from multiple vendors. We then analyze the security of the model in Section 5 and conclude this paper in Section 6.

## 2. Online Application Store

**Figure 1:**
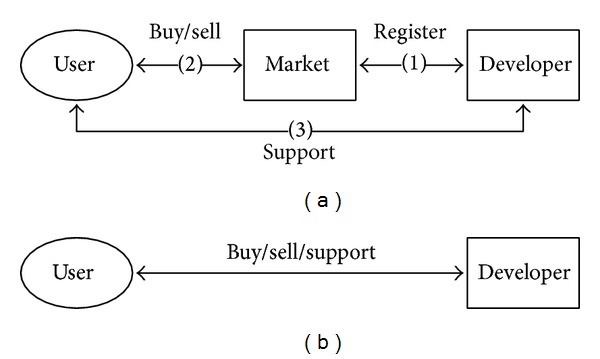
Traditional distribution—(a) individual markets; (b) direct from developers.

Commercial consumer software was traditionally distributed as a package through offline markets. Users purchased an application from either (a) individual markets or (b) developers directly, as shown in Figure 1. When digital download services were introduced, users could still purchase from either source and receive support for that application by directly contacting the developer.

Conventional markets, however, must address the following two issues:* license management* and* software installation*. For license management, users purchasing from a web-market could download the application directly from the specific website. For authorization, users received license codes through emails or receipts. Users had to keep or request new license codes from distributors when they were required to reinstall the app.

The mobile handset market, in addition to the PC market, also provided digital download services. For example, legacy mobile devices, such as those based on* Palm OS* and* Windows CE*, widely used until the late 2000s, enabled users to install any application they chose to their devices.

**Figure 2:**
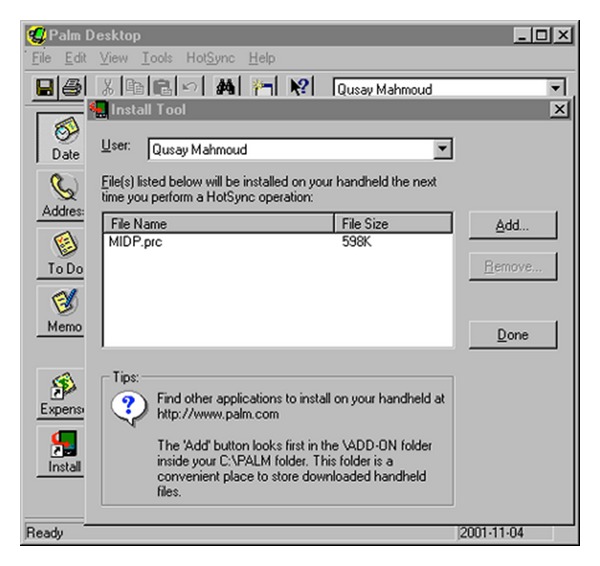
Application management in Palm OS. Legacy devices needed to connect to a PC to install mobile applications.

The installation process, however, was not convenient.  Figure 2 shows an example of installing an application on a* Palm OS*-based device. To install an application, users had to manage a desktop application that synchronized with the mobile device.

Although later Wi-Fi-enabled devices could download and install applications without desktop tools, the purchase and authorization process remained the same as shown in Figure 1. Users still managed their license codes themselves.

### 2.1. Online Application Stores

**Figure 3:**
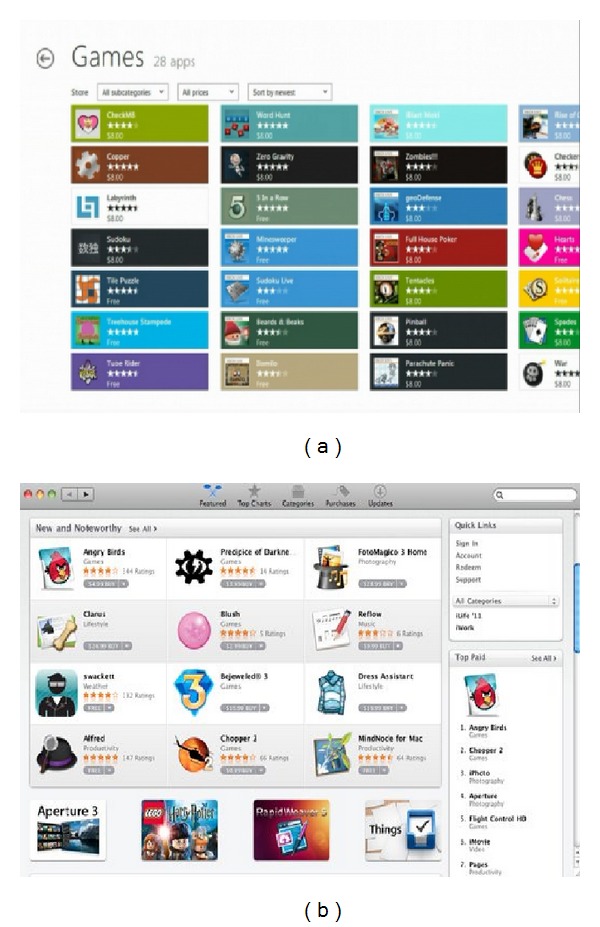
(a) Windows marketplace (b) Mac Appstore.

Online application stores (OASs) provide users with easier license and application code management. When a user purchases an app from an OAS, it requires only one click to install, reinstall, or update the app. In fact, the market share of* Palm OS*,* Windows CE*, and even* Symbian OS* quickly decreased once Apple launched the* Appstore* for* iPhone*. OASs are not only used with mobile devices. PC environments, including Windows and Mac OS X, and software distributors such as Amazon are rapidly deploying market services, as shown in Figure 3.

**Figure 4:**
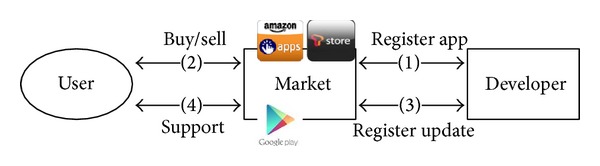
Online application distribution.

Multiple OASs are often preinstalled or installed by users on their devices and systems. By connecting to an OAS, a user can easily purchase applications. When a user needs support, they can easily get updates from their application provider, as shown in Figure 4.

### 2.2. Types of OAS User Registration

We separate OASs into the three groups discussed in [1]: OAS from OS holder (Type 1), OAS from Content distributor (Type 2), and OAS from mobile carriers (Type 3).

- Type 1: the store application is preinstalled on the device. Users register their accounts with the service. To purchase applications, users must also register their billing information. Depending on the billing information or location information, the store can provide localized services. Microsoft's* Windows Store*, Apple's* Appstore*, the Ubuntu* Software Center*, and* Google Play* are examples.
- Type 2: users manually install the store application on their device. Users register their accounts. To purchase applications, users must also register their billing information. Stores authenticate users with the billing information.* Amazon Appstore* and* Steam Online* are examples.
- Type 3: mobile carriers/manufacturers preinstall their own OASs on the device. Mobile users are already registered through their carrier subscription information. The store verifies the information from the USIM in the device. Only subscribed devices can use the service. Users must be connected to the cellular network.

## 3. Application Management from Multiple OASs

In this section, we discuss issues with application management from multiple OASs and extend the PAS introduced in [1] to overcome such issues.

### 3.1. Application Management from Multiple OASs

#### 3.1.1. Software License Check Problem

Software piracy has long been a serious security problem. Many proposals [2–4] have attempted to prevent this problem by the use of a software license confirmation. They focus on the verification of the validity of the software license from the original source.

**Figure 5:**
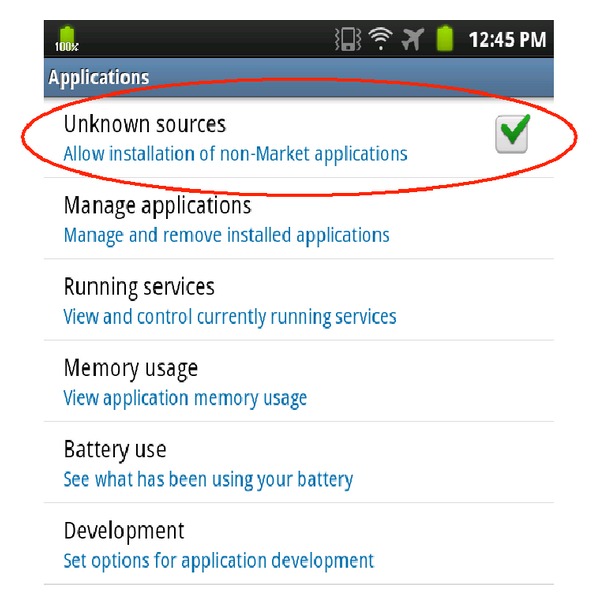
Android OS allows unauthorized applications.

For example, the Android system enables anyone to develop and distribute Android software. It uses the Android application package file (APK) format to distribute and install applications and middleware to the device. Although the Android system initially demands that all applications be signed by the application developer to ensure the trust of the applications, installing unauthorized applications manually is also allowed, as shown in Figure 5. This openness permits the illegal distribution of cracked applications and malware (Graham Cluley, “Android malware poses as Angry Birds Space game,” NakedSecurity, SophosLab April 12, 2012) to the Android system [5].

Therefore, many researchers have focused on prevention mechanisms against such threats [6–9], including online authentication. Such authentication systems, however, can decrease the availability of applications.

While many vendors do deploy online authentication systems, Type 3 distributors generally use an authentication process via a wireless network. For example, Korean local distributors, including* SKT T-store* and* KT Olleh market*, commonly utilize users' subscribed information in the USIM over the cellular network. When a user launches an application, they must be connected to the network. Those who are not connected to the network via a cellular connection fail to be authenticated.

#### 3.1.2. Software Support without Original OAS

In the traditional application distribution environment shown in Figure 1, stores only provide applications to users. Users then contact the developers directly for support. Although this is not a very convenient process, once a user has purchased an application, they can obtain continued support from the developer.

In contrast, in the current distribution process shown in Figure 4, OASs not only sell applications but also provide support to users. Although such a mechanism brings huge convenience to users, they must maintain their connection to the store to receive this support. A user who cannot contact the store will fail to get further support and must purchase the same application again, from a different store that he can contact. This generally occurs for Types 2 and 3 cases, where the OAS only provides service for the localized domain.

#### 3.1.3. OAS Management Problem

OAS users can purchase software from multiple OASs, as shown in Section 2.2. This can cause problems with verifying the license. Although allowing multiple OASs in a device allows users to choose their preferred service, any OAS that a user contacts can manage the software. Whereas legacy distribution systems allow users to get software support, regardless of where the application was purchased, OAS users can only get support from the OAS from whom they made their purchase.

**Figure 6:**
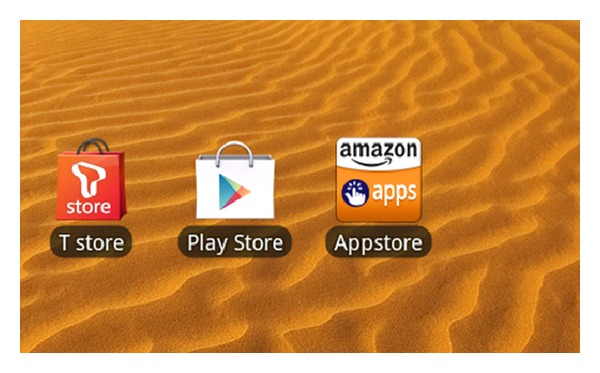
An example of various OASs in one mobile device:* SKT T-store* (Type 3),* Google Play store* (Type 1), and* Amazon Appstore* (Type 2).

Therefore, OAS users who purchase apps from multiple OASs must manage multiple OAS systems in the device, as shown in Figure 6. This increases the management overhead, especially to mobile device users.

### 3.2. PAS Model

In order to resolve the issues discussed above, we propose a PAS model. This enables users who have already purchased applications to receive support when they change their status or cannot reach the original OAS, temporarily or permanently [1]. We define the PAS as a trusted entity that stores users' purchase records. We have limited the functionality of the PAS model to the mobile environment.

## 4. Improved PAS Model

Maintaining an additional trusted entity for the PAS could increase the management overhead. In this paper, therefore, we extend the model by adding a PKI feature and modify the PAS as a part of this service. This does not require an additional entity, and hence there are no additional management issues. When a user purchases an application from OAS_1_, OAS_1_ stores the user's purchase record. At a later time, if a user loses contact with OAS_1_, he can obtain support from OAS_2_ by providing this proof of purchase. We assume that a user is always registered with at least one service.

### 4.1. Improved System Model

**Figure 7:**
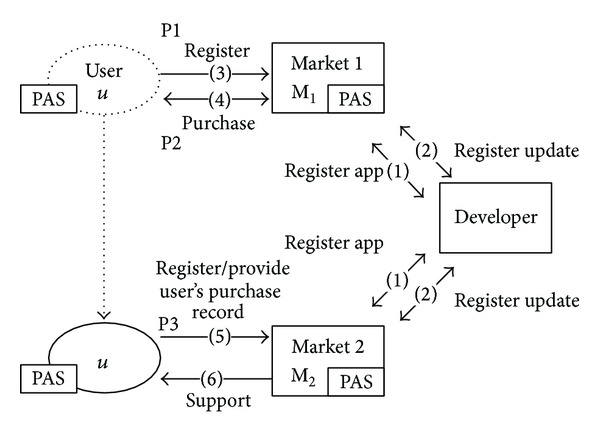
System model.

We assume the following system model, illustrated in Figure 7. A developer provides applications to the OASs. A user, *U*, purchases applications from the OASs. The OASs share their public keys, pk_*j*_, 0 ≤ *j* ≤ *n*, where *n* is the ID of the OAS. Stores (OAS_1_ and OAS_2_) have a secure association. OASs also share the seed secret, *s*. Developers always register (1) and update (2) their applications. *U* must first register himself to an OAS, say, OAS_1_. (3) After successful registration, *U* may purchase multiple applications from OAS_1_. (4) OAS_1_ stores *U*'s information using the PAS. When *U* has a status change, he may request to update his registration to a newly connected store, OAS_2_ (5). OAS_2_ provides services after validating *U* (6).

### 4.2. P1: Initial User Registration Phase

The user registration process is initiated when a user first registers with a specific store. Let a user, *U*, register with store OAS_1_.

**Figure 8:**
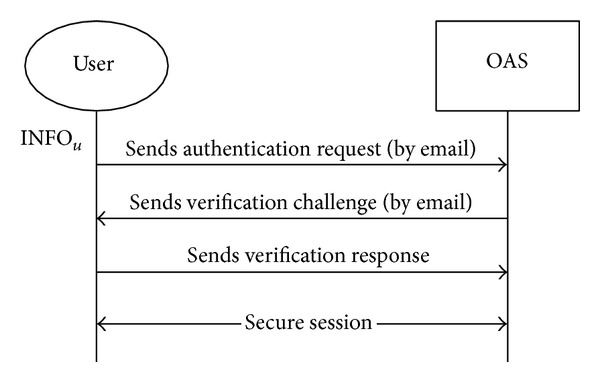
Checking* UAddr* by email verification.

When *U* requests their registration to OAS_1_, OAS_1_ establishes a secure channel with *U*. We assume that email is used to establish the secure channel, as is used by many Internet services.  Figure 8 shows an example of this process.

**Figure 9:**
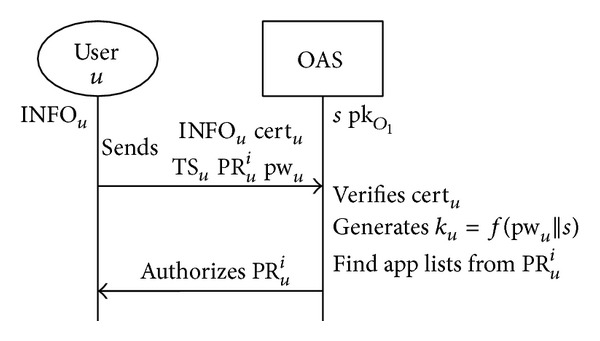
P1: Registration phase.

The Registration phase, P1 in Figure 7, registers *U* to OAS_1_ as shown in Figure 9. INFO_*U*_ denotes the user purchase record stored by the OAS. When a user requests support from OAS_1_, the store verifies INFO_*U*_. INFO_*U*_ includes the elements in Table 1.

When *U* and OAS_1_ establish a secure channel, *U* selects and sends PWD to OAS_1_. OAS_1_ gathers INFO_*U*_ and generates cert_*U*_ as follows:

(1) certU=sign⁡skO1{h(AddrUP)||h(pwU)||⋯(opt)    ⋯||h(TS)||h(PRU)},

where *sk*_*O*_1__ is a private key of OAS_1_ and sign⁡_*k*_{*m*} denotes a signature of *m* signed by *k*. PR_*U*_ = enc_*k*_*U*__{App_*i*_}, where *i* is the purchased app ID and *k*_*U*_ = *f*(pw_*U*_||*s*), where *f*(*m*) is a key generation function with input *m* and *s* is the seed secret of the OASs. opt denotes optional information for deployment.

OAS_1_ then sends TS, PR_*U*_, and cert_*U*_ to *U*. *U* stores cert_*U*_, PR_*U*_, and TS.

### 4.3. P2: Purchase Phase

The purchase phase, P2 in Figure 7, is invoked when *U* purchases applications from OAS_1_.

When *U* purchases App_*i*_ from OAS_1_, OAS_1_ updates PR_*U*_. In the first step, OAS_1_ decrypts PR_*U*_ with *k*_*U*_ and adds APP_*i*_ to the application list. If *s* is updated to *s* ^new^, OAS_1_ generates a new *k*_*U*_^new^ = *f*(pw_*U*_ | |*s* ^new^). Then, PR_*U*_^new^ is generated by encrypting the updated application list using *k*_*U*_^new^.

Finally, OAS_1_ sends PR_*U*_^new^ to *U*, where it is also stored.

### 4.4. P3: Purchase Authentication Phase

The purchase authentication phase, P3 in Figure 7, is invoked when *U* contacts a new OAS, one from whom he did not purchase the application. We consider the case where *U* requests support from OAS_2_. We assume that *U* registers himself with OAS_2_, using the user registration phase, or temporarily contacts OAS_2_ and then requests support for an application already purchased from OAS_1_.

#### 4.4.1. Step  1: Check User's Registration Information

To verify *U*'s purchase record, OAS_2_ checks *U*'s registration information Addr_*U*_, as shown in Figure 8. Through a secure channel, *U* requests the purchase authentication from OAS_1_ and sends INFO_*U*_ with pw_*U*_, cert_*U*_, TS_*U*_, and PR_*U*_ to OAS_2_. OAS_2_ then verifies cert_*U*_ with OAS_1_'s public key pk_*O*_1__. After verifying *U*'s registration information, OAS_2_ generates *k*_*U*_ with pw_*U*_ and *s*. OAS_2_ then decrypts *U*'s purchase record, PR_*U*_, with *k*_*U*_.

#### 4.4.2. Step  2: User Authorization

The processes are slightly different depending on the user's status. In this paper, we show two cases: *U* temporarily uses a roaming service and *U* permanently changes his OAS.

*Case A*: *U Temporarily Uses a Roaming Service*. When *U* connects to OAS_2_ as a roaming service, OAS_2_ grants temporary authorization to *U*. *U* still has his original PR_*U*_, INFO_*U*_, and Cert_*U*_, and OAS_2_ does not send a new certificate. OAS_2_ has access to the billing information of *U* and can request payment. The authorization remains valid for a specific time period; for example, OAS_2_ can authorize *U* for one day. 

*Case B: U* *Permanently Changes His OAS*. When *U* permanently changes from OAS_1_ to OAS_2_, Cert_*U*_ from OAS_1_ is revoked and OAS_2_ issues a new Cert_*U*_^new^ to *U*. INFO_*U*_ is updated. For example, *U* may have a new Addr_*U*_^*S*^. If the user keeps his old email, Addr_*U*_^*P*^ does not change. If the user connects using the same device, PN_*U*_ remains unchanged. TS_*U*_ is updated. *U* receives INFO_*U*_^new^, Cert_*U*_ and stores them with PR_*U*_. OAS_2_ also stores the information. By this process, OAS_2_ can bill *U*.

## 5. Security Analysis

In this section, we show that the security of the design satisfies standard security requirements and also show that the design is secure against possible attack. We assume OAS_*i*_, where *i* is the ID of the OAS, can be trusted. Performance is not an issue in this paper and depends upon the actual deployment case.

### 5.1. Security Requirements

The following are the security requirements for the PAS model.

- Nonrepudiation: the user should not be able to claim that his records are invalid.
- Authentication: the distributor must be able to validate the user's request.
- Privacy: the distributor can only know the user's information after the user is approved.

The handling of malicious applications in the store is not the focus of this paper.

### 5.2. Nonrepudiation

pw_*U*_ is chosen by *U*, and *U* does not know the *k*_*U*_ generated from pw_*U*_. Since OAS_2_ can request information about PR_*U*_ only when *U* requests a service, repudiation from *U* can be prevented.

### 5.3. Authentication

Cert_*U*_ enables OAS_*i*_ to check the validity of *U*. Since only a valid OAS_*i*_ can generate a Cert_*U*_, using the private key *sk*_*O*_*i*__, a malicious user or other attacker cannot forge or abuse it.

### 5.4. Privacy

Without pw_*U*_, OAS_*i*_ cannot access the application list in PR_*U*_. OAS_*i*_ can only generate the *k*_*U*_ that decrypts PR_*U*_ to see the application list when *U* sends pw_*U*_.

*U* can replace pw_*U*_ at any time. Although a specific OAS_*i*_ can see the application list if a user chooses to use a temporary roaming service, it will not be aware of any future changes to pw_*U*_.

Only hashed user information from Table 1 is stored in Cert_*U*_. Thus, an unapproved OAS_*i*_ cannot know the information before *U* provides them with access.

### 5.5. Security against Possible Attack Scenarios

We assume that a malicious user Eve, *E*, could try to obtain support from a market without any purchase record. *E* could try the following scenarios.

#### 5.5.1. Fraudulent User Tries to Get Authorization Illegally

*E* impersonates *U*. In this case, where the attacker impersonates a legal user, *E* would require INFO_*U*_, including Addr_*U*_, to impersonate *U* in OAS_1_ or OAS_2_.

*E* would not, however, be able to enter Addr_*U*_ during the PAS registration phase described in Section 4.4.1. Securing *U*'s email account is not the focus of this paper.

Even when *E* compromises *U*'s device and extracts INFO_*U*_ and Cert_*U*_, *E* still does not know the password pw_*U*_. Deploying the PAS model, OAS_*i*_ can limit the number of password attempts. For example, if several invalid password entries are attempted, OAS_*i*_ can temporarily place *U*'s account on hold. In such a case, *U* would have to contact the service by phone or physical mail. We do not show the details of this process in this paper.

#### 5.5.2. Forged Purchase Record

The malicious user *E* could forge his own purchase record PR_*E*_. In this case, *E* would have to be able to modify PR_*E*_ in the PAS. For this to succeed, *E* would have to know *s* in order to generate *k*_*E*_ and then generate Cert_*E*_. This is impossible without knowing *sk*_OAS_*i*__.

## 6. Conclusion

With OASs becoming the main channel of software distribution, software license authentication issues present a potential problem when using multiple OASs. We have discussed possible issues from using multiple OASs and proposed an improved PAS model that reduces management overheads without any additional entity, while still allowing users to obtain support from multiple OASs. We refined our model to support a temporary roaming situation, as well as a permanent OAS change. We described the security of the proposed model.

Our design shows not only the technical availability of ongoing benefits to users, but also a possible business model for OASs.

## Figures and Tables

**Table 1:** INFO_*U*_ elements.

Element	Description
Addr_*U*_^*P*^	User's primary contact information (e.g., email address)
Addr_*U*_^*S*^	User's supplementary information (e.g., phone number; optional)
PN_*U*_	Device information (optional)
CN_*U*_	Country code (optional)
CR_*U*_	Carrier code (Type 3)
TS	Timestamp
pw_*U*_	Passcode for the registration
PR_*U*_	Purchase record of *U*
